# MRI-based habitat radiomics for predicting WHO/ISUP nuclear grade in clear cell renal cell carcinoma

**DOI:** 10.3389/fonc.2026.1751579

**Published:** 2026-01-30

**Authors:** Naijing Shi, Cong Zhang, Xinyi Li, Mohan Hao, Chong Liu

**Affiliations:** 1 Department of Radiology, The First Central Hospital of Baoding, Baoding, Hebei, China; 2 Graduate School, Chengde Medical University, Chengde, Hebei, China

**Keywords:** clear cell renal cell carcinoma, habitat, peritumoral, radiomics, WHO/ISUP nuclear grade

## Abstract

**Objective:**

This study aimed to develop an explainable fusion model that integrates intratumoral, peritumoral, and habitat features derived from MRI to evaluate its feasibility for predicting the WHO/ISUP nuclear grade of clear cell renal cell carcinoma (ccRCC).

**Methods:**

We retrospectively enrolled 154 patients with pathologically confirmed ccRCC and partitioned them into a training set (n = 108) and an independent test set (n = 46). On contrast-enhanced T1-weighted images, regions of interest were manually delineated layer-by-layer along the tumor margin and expanded outward by 1 mm, 2 mm, 3 mm, 4 mm and 5 mm to derive peritumoral regions. Tumor habitat regions were identified using the K-means clustering algorithm. After extraction and selection of radiomic features, radiomics and habitat models were constructed using five machine learning algorithms. These effective features were then integrated into a nomogram. Model performance was assessed by plotting receiver operating characteristic (ROC) curves and calculating the area under the curve (AUC). Model calibration and clinical utility were evaluated using calibration curves and decision curve analysis (DCA). Model interpretability was enhanced by employing Shapley Additive exPlanations (SHAP).

**Results:**

Three habitat subregions were identified within tumors. The integrated habitat region(Habitat) model demonstrated the highest performance among the evaluated habitat models, with AUCs of 0.894 and 0.877 in the training and test sets, respectively. The Peri2mm model achieved AUCs of 0.884 and 0.839, outperforming other peritumoral ranges. Therefore, the 2-mm peritumoral margin was considered a potentially optimal analysis range in this cohort.When the integrated habitat region signature was combined with intratumoral features, 2-mm peritumoral features and the independent clinical predictor (corticomedullary enhancement level) in a nomogram, predictive performance was further improved, achieving AUCs of 0.934 and 0.912. SHAP bee swarm and force plots provided intuitive visualization of the habitat model’s decision-making process.

**Conclusion:**

The nomogram, which integrates intratumoral, peritumoral and habitat radiomic features derived from MRI, demonstrated excellent performance for noninvasive preoperative prediction of WHO/ISUP nuclear grade in ccRCC and holds promise as an adjunctive tool for individualized therapy planning and prognostic assessment. However, its clinical application requires further external validation.

## 1 Introduction

Renal cell carcinoma (RCC) is the 14th most prevalent cancer globally, accounting for more than 400,000 new diagnoses in 2020 (1, 2). Its global incidence has continued to rise in recent years, posing a substantial public-health burden. Clear cell renal cell carcinoma (ccRCC) constitutes the predominant histologic subtype of RCC, representing approximately 80% of all cases (1). Extensive research has established that pathological tumor nuclear grade is closely correlated with prognosis in ccRCC patients (3). The 2016 World Health Organization/International Society of Urological Pathology (WHO/ISUP) nuclear grading system has been widely adopted in clinical practice (4). This system stratifies tumors from grade I to IV, with grades I–II considered low grade and grades III–IV high grade. High-grade ccRCCs generally exhibit greater aggressiveness and a higher propensity for recurrence, resulting in poorer outcomes (3, 5). Consequently, accurate preoperative assessment of tumor nuclear grade is critical for devising individualized treatment strategies.

At present, preoperative nuclear grading primarily depends on percutaneous biopsy. This invasive procedure carries risks such as hemorrhage and needle tract seeding. Moreover, intratumoral heterogeneity and sampling limitations can lead to misclassification of nuclear grade (6, 7). Magnetic resonance imaging (MRI), with its superior soft-tissue contrast, absence of ionizing radiation, and multiparametric capability (8), plays a central role in the diagnostic evaluation and management of ccRCC. Nevertheless, conventional MRI has limitations such as interobserver variability and limited reproducibility. Thus, there is an urgent clinical need to develop a noninvasive, accurate method for preoperative nuclear grading to better inform precision treatment decisions.

Radiomics enables precise quantitative assessment of tumor biological characteristics through the high- throughput extraction and analysis of quantitative imaging features, integrated with artificial intelligence algorithms such as machine learning and deep learning (9). Currently, MRI-based radiomics has demonstrated considerable promise in ccRCC for differential diagnosis (10), nuclear grading (11, 12), and prognostic assessment (13). However, most existing investigations have focused predominantly on the intratumoral region, with relatively fewer studies addressing the peritumoral region. The peritumoral compartment, an integral component of the tumor microenvironment, harbors abundant stromal cells and cytokines that play pivotal roles in tumor initiation and progression (14, 15). Furthermore, accumulating evidence indicates that peritumoral imaging features can materially enhance model performance (16–18). Conventional radiomics approaches typically treat intratumoral and peritumoral regions as homogeneous wholes, thereby overlooking spatial heterogeneity shaped by cellular subpopulations, vascular architecture, and the immune microenvironment. To overcome this limitation, habitat imaging technology has been developed. This technique partitions the tumor into subregions composed of voxels with similar imaging signatures via clustering algorithms (19), thereby enabling fine-grained quantification of intratumoral heterogeneity. Each habitat subregion embodies distinct biological properties, reflecting differences in angiogenesis, cellularity, metabolic activity, and gene expression, and thus reveals the spatial heterogeneity and biological complexity of the tumor (20). Habitat radiomics has shown great promise in studies of glioma (21), oral squamous cell carcinoma (14), and breast cancer (22). However, MRI-based habitat radiomics for predicting the ccRCC WHO/ISUP nuclear grade remains unexplored.

In this study, we aimed to construct a nomogram based on contrast-enhanced T1-weighted images by integrating clinical variables with intratumoral, peritumoral and habitat radiomic features to predict WHO/ISUP nuclear grade in ccRCC. In addition, Shapley Additive Explanations (SHAP) analysis was incorporated to enhance model transparency and interpretability, thereby helping to support clinical decision-making.

## 2 Materials and methods

### 2.1 Patients

**Figure 1:**
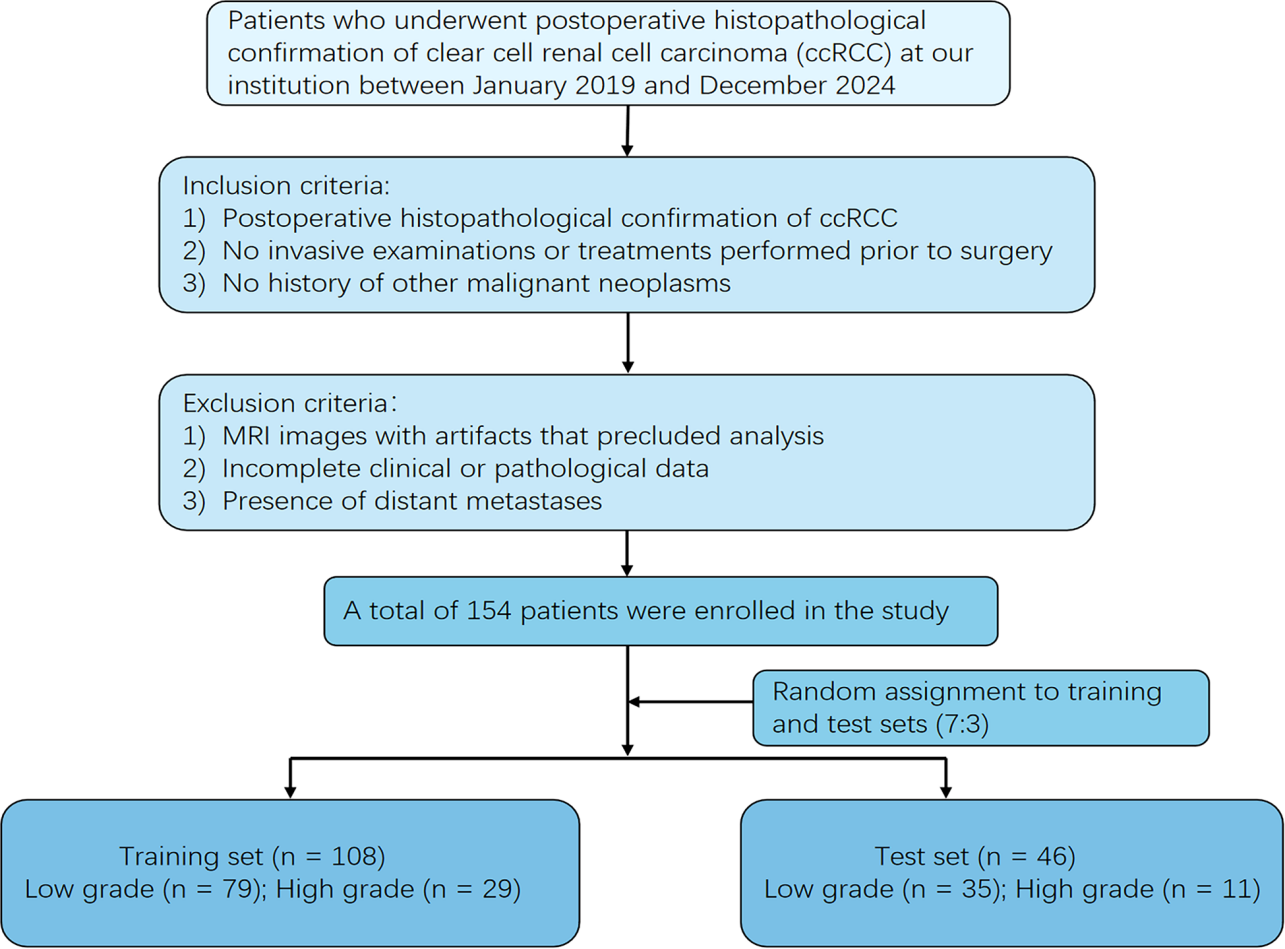
Flowchart of patient selection in this study.

This investigation employed data from 154 patients with pathologically confirmed ccRCC who underwent preoperative MRI at The First Central Hospital of Baoding between January 2019 and December 2024 (training set, n = 108; test set, n = 46). Inclusion criteria were: (1) postoperative histopathological confirmation of ccRCC; (2) no invasive examinations or treatments performed prior to surgery; and (3) no history of other malignant tumors. Exclusion criteria were: (1) MRI images with artifacts that precluded analysis; (2) incomplete clinical or pathological data; and (3) presence of distant metastases. A 7:3 ratio was used to randomly assign the patients to the training and testing sets (**Figure 1**). Ethical approval for the study (Ethics Approval No. Kuai [2025] 017) was granted by our hospital’s ethics committee with a waiver of informed consent.

### 2.2 MRI acquisition

All MRI acquisitions were carried out on a Philips Achieva TX 3.0-T machine. During scanning, patients were placed in the supine position and asked to hold their breath. A 16-channel phased-array body coil was used to fully cover the kidneys and lesion region. Contrast-enhanced T1-weighted imaging sequence parameters were: TE 1.7 ms, TR 5.8 ms, interslice gap 0.78 mm, slice thickness 5 mm, and FOV 220 mm × 189 mm. Contrast administration consisted of gadopentetate dimeglumine (Gd-DTPA; Bayer Healthcare Pharmaceuticals, Germany), administered at a dose of 0.1 mmol/kg via power injector at 2 ml/s, followed immediately by a 20 ml saline flush at the same rate. The corticomedullary phase images were acquired at 15–20 s postinjection.

### 2.3 Clinical and imaging data collection

We collected baseline clinical data from the hospital electronic medical record system, which encompassed age, sex, history of hypertension, smoking history, history of diabetes, and hematuria. Two abdominal radiologists with six years of experience conducted a blinded independent review of the imaging features. Evaluated imaging variables included maximum tumor diameter (mean of three measurements), tumor location, lesion margin, pseudocapsule, renal vein invasion, renal sinus involvement, intratumoral vessels, cystic necrosis, and corticomedullary enhancement level. Corticomedullary-phase enhancement was evaluated qualitatively by visual comparison with the contralateral normal renal cortex on the same imaging plane and classified into three grades: high, iso, and low enhancement. Discrepancies were resolved by a senior expert with more than 15 years of experience in medical imaging diagnostics.

### 2.4 Image preprocessing and tumor segmentation

**Figure 2:**
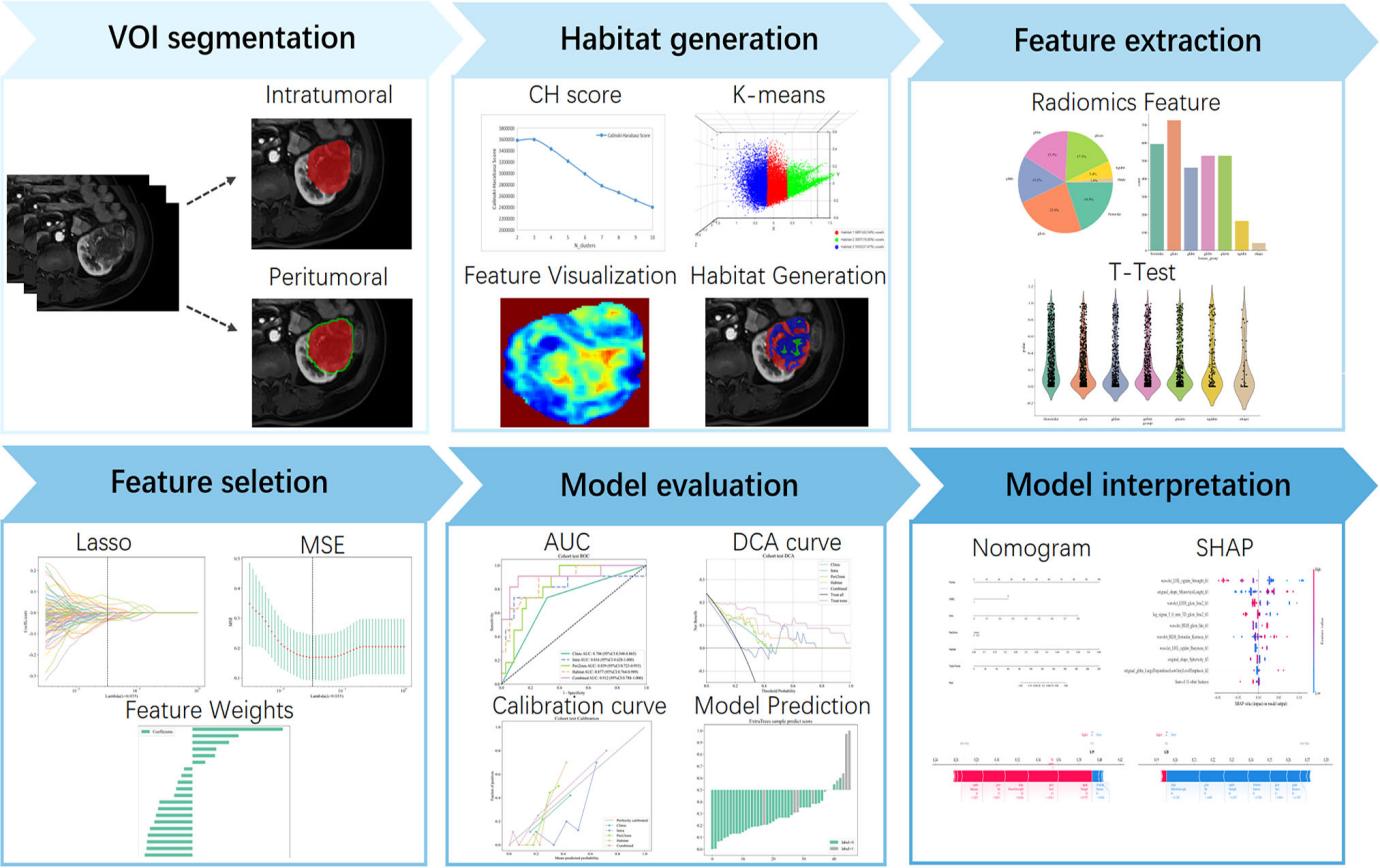
Overall workflow of this study.

All DICOM images retrieved from the Picture Archiving and Communication System were preprocessed to harmonize acquisition differences and ensure feature comparability. Preprocessing steps included N4 bias-field correction, voxel resampling to isotropic 1 × 1 × 1 mm³, and intensity normalization. Two radiologists manually delineated the tumor boundary slice-by-slice on axial contrast-enhanced T1-weighted images using ITK-SNAP (version 3.8.0) to generate three-dimensional volumes of interest (VOIs). All delineations were independently reviewed by a senior radiologist, and final decisions in cases of disagreement were made by this reviewer. To assess interobserver reproducibility, 30 cases were randomly re-segmented two weeks later, and intraclass correlation coefficients (ICC) were computed. We defined features with an ICC value ≥ 0.75 as having adequate inter-rater reliability. The overall study design and workflow are summarized in **Figure 2**.

### 2.5 Peritumoral and habitat region generation

**Figure 3:**
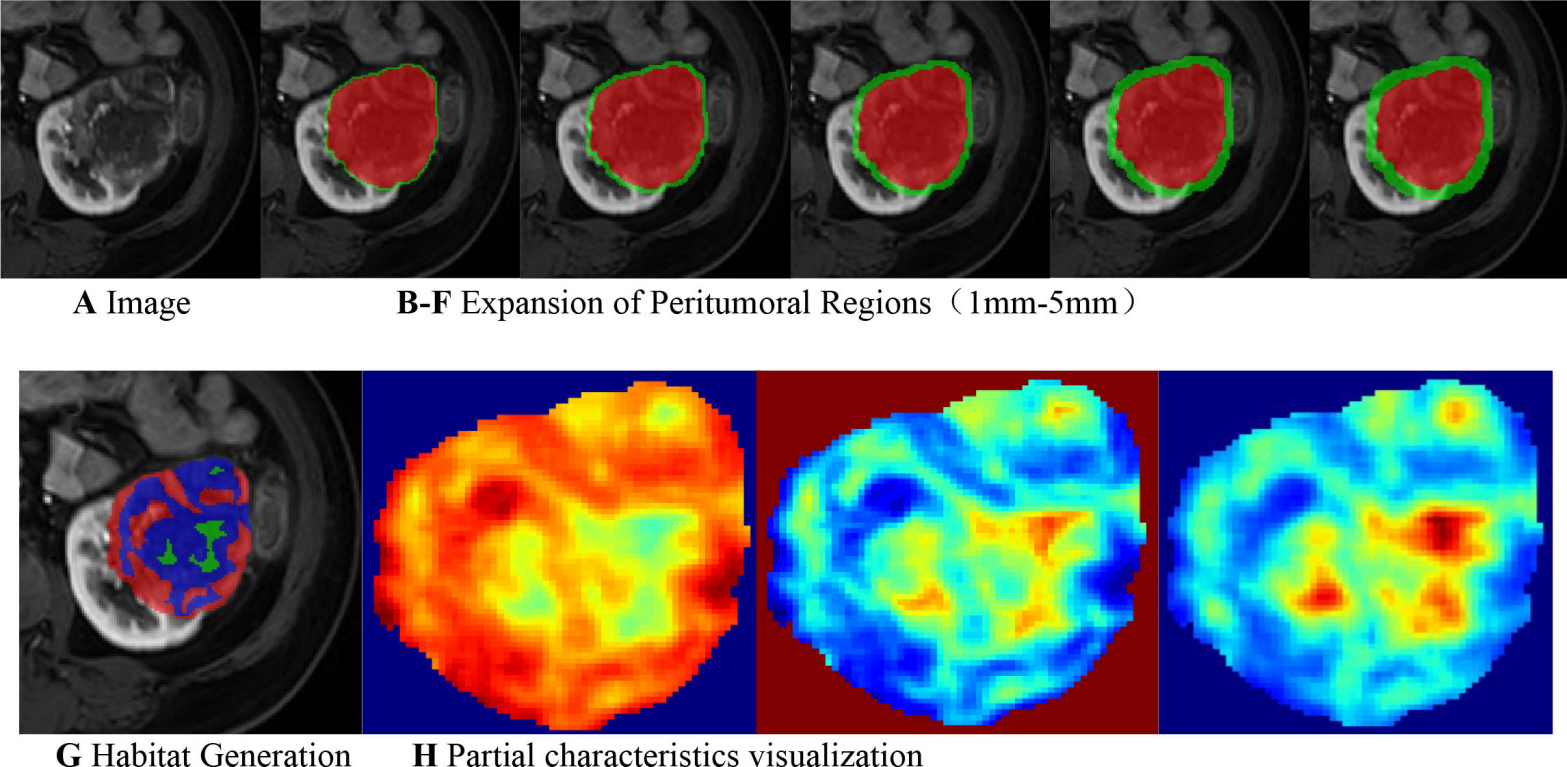
**(A)** Original image. **(B–F)** Tumor peritumoral region expansion, red represents the intratumoral region, and green represents the peritumoral region, with expansion of the peritumoral margin at 1 mm, 2 mm, 3 mm, 4 mm, and 5 mm. **(G)** Habitat Generation. **(H)** Partial characteristics visualization(original_firstorder_Entropy,original_glcm_Imc1,and original_glcm_InverseVariance).

Peritumoral rings were generated by automatically expanding the delineated tumor margin outward by 1 mm, 2 mm, 3 mm, 4 mm, and 5 mm. The optimal peritumoral extent for subsequent analysis was selected based on comparative predictive performance across these candidate ranges. To quantify intratumoral heterogeneity, we applied a sliding window (5 × 5 × 5) within the tumor VOI to extract 19 local features voxel-by-voxel. K-means clustering was then performed to identify tumor subregions composed of voxels with similar imaging signatures. The optimal number of clusters (k) was determined by maximizing the Calinski–Harabasz (CH) index in the range 2–10. **Figure 3** shows schematics of peritumoral expansion and clustering results of tumor regions.

### 2.6 Feature extraction and selection

Quantitative imaging features were derived from each region using the PyRadiomics package (version 3.0.1), covering first-order statistics, shape descriptors, and texture features. Because unsupervised clustering can yield missing habitat subregions for some samples, missing habitat features were imputed using a k-nearest neighbors (KNN) algorithm.

To improve feature robustness, a multistep feature selection pipeline was implemented. First, features with poor interobserver reproducibility (ICC < 0.75) were excluded. Notably, the ICC was not applied to unsupervised habitat features, which were therefore exempt from this step. Remaining features were standardized by Z-score normalization. Depending on distributional properties, univariate filtering was conducted utilizing either independent-samples t tests or the Mann-Whitney U test for the identification of discriminatory features between the groups. Next, we calculated Pearson correlation coefficients and subsequently eliminated features that demonstrated high intercorrelation (|r| > 0.9). Finally, the least absolute shrinkage and selection operator (LASSO) regression with ten-fold cross-validation was employed to select the optimal subset of predictive features.

### 2.7 Model development

Independent clinical predictors demonstrating significant correlations with ccRCC nuclear grade were identified through univariate and multivariate logistic regression. These predictors were then used to construct a clinical prediction model (Clinic). Radiomic models were developed for multiple regions using five machine learning algorithms, including logistic regression (LR), support vector machine (SVM), random forest (RF), extremely randomized trees (ExtraTrees), and Light Gradient Boosting Machine (LightGBM). Regional models included intratumoral (Intra), peritumoral at 1–5 mm (Peri1mm–Peri5mm), habitat subregions (HabitatHx), and the integrated habitat region(Habitat). All models underwent hyperparameter tuning via five-fold cross-validation to enhance stability. The algorithm that achieved the highest area under the curve (AUC) on the test set was selected as the optimal classifier for each region. Furthermore, clinical variables were integrated with the optimal intratumoral, peritumoral, and habitat models to construct a nomogram. The nomogram provides a visual, quantitative tool for preoperative nuclear-grade prediction and therefore has the potential to support clinical decision-making.

### 2.8 Model evaluation and explainability analysis

Model discrimination was assessed by receiver operating characteristic (ROC) curve analysis, with computation of sensitivity, specificity, accuracy, and AUC for a comprehensive evaluation. DeLong’s test was used to compare AUC values across different models to assess statistical significance. Calibration performance was examined with calibration curves, and clinical utility was appraised by decision curve analysis (DCA). To elucidate the decision logic of the habitat model, we performed explainability analyses at both global and individual levels using SHAP. A SHAP swarm plot was used to visualize overall feature importance. SHAP force plots were employed to decompose the predictions for individual samples.

### 2.9 Statistical analysis

All statistical analyses were performed in Python (version 3.7.0). Continuous variables were compared between groups using either the independent samples t-test or the Mann-Whitney U test, depending on their distributional characteristics. Categorical variables were compared using the chi-square test or Fisher’s exact test as appropriate. A significance threshold of p-value < 0.05 was adopted for all two-tailed tests.

## 3 Results

### 3.1 Patient characteristics

Our study enrolled a total of 154 participants (105 males, 49 females, mean age 59.51 ± 11.09 years). In the training set, 79 patients were classified as low grade and 29 as high grade, while in the test set, 35 were low grade and 11 were high grade. In both the training and test sets, statistically significant differences (*P* < 0.05) were observed between the low- and high-grade groups in terms of tumor maximum diameter, renal sinus involvement, renal vein invasion, intratumoral vessels, and corticomedullary enhancement level (**Table 1**).

**Table 1:** Comparison of clinical features between the training set and test set.

Characteristic	Training set(n=108)		p-value	Test set(n=46)		p-value
	Low-grade	High-grade		Low-grade	High-grade	
	(n=79)	(n=29)		(n=35)	(n=11)	
Age	60.076 ± 10.793	63.862 ± 8.039	0.088	54.514 ± 12.832	59.818 ± 9.918	0.216
Maximum Tumor Diameter	3.756 ± 2.188	6.928 ± 2.342	<0.001	3.583 ± 1.450	8.182 ± 2.297	<0.001
Renal sinus involvement	0.241 ± 0.430	0.759 ± 0.435	<0.001	0.143 ± 0.355	0.818 ± 0.405	<0.001
Sex			0.788			0.464
Female	23(29.114)	7(24.138)		16(45.714)	3(27.273)	
Male	56(70.886)	22(75.862)		19(54.286)	8(72.727)	
Tumor location			1.000			0.066
Left	37(46.835)	13(44.828)		16(45.714)	1(9.091)	
Right	42(53.165)	16(55.172)		19(54.286)	10(90.909)	
Lesion margin			<0.001			0.052
Clear	59(74.684)	10(34.483)		26(74.286)	4(36.364)	
Unclear	20(25.316)	19(65.517)		9(25.714)	7(63.636)	
Smoking history			1.000			0.199
Absent	53(67.089)	20(68.966)		28(80.000)	6(54.545)	
Present	26(32.911)	9(31.034)		7(20.000)	5(45.455)	
History of hypertension			1.000			1.000
Absent	38(48.101)	14(48.276)		15(42.857)	5(45.455)	
Present	41(51.899)	15(51.724)		20(57.143)	6(54.545)	
History of diabetes			0.622			0.762
Absent	60(75.949)	24(82.759)		29(82.857)	8(72.727)	
Present	19(24.051)	5(17.241)		6(17.143)	3(27.273)	
Hematuria			0.501			0.427
Absent	71(89.873)	24(82.759)		31(88.571)	8(72.727)	
Present	8(10.127)	5(17.241)		4(11.429)	3(27.273)	
Pseudocapsule			1.000			1.000
Absent	39(49.367)	15(51.724)		17(48.571)	6(54.545)	
Present	40(50.633)	14(48.276)		18(51.429)	5(45.455)	
Intratumoral vessels			<0.001			<0.001
Absent	59(74.684)	9(31.034)		31(88.571)	3(27.273)	
Present	20(25.316)	20(68.966)		4(11.429)	8(72.727)	
Renal vein invasion			<0.001			0.002
Absent	75(94.937)	20(68.966)		34(97.143)	6(54.545)	
Present	4(5.063)	9(31.034)		1(2.857)	5(45.455)	
Cystic necrosis			0.092			0.281
Absent	32(40.506)	6(20.690)		11(31.429)	1(9.091)	
Present	47(59.494)	23(79.310)		24(68.571)	10(90.909)	
Corticomedullary Enhancement Level			<0.001			0.038
Lower than renal parenchyma	21(26.582)	20(68.966)		11(31.429)	8(72.727)	
Higher than or equal to renal parenchyma	58(73.418)	9(31.034)		24(68.571)	3(27.273)	

### 3.2 Feature selection and model construction

Univariate and multivariate logistic regression analysis (**Table 2**) identified corticomedullary phase enhancement as a significantly associated independent factor of ccRCC nuclear grade (*P* < 0.001). A clinical model was then constructed accordingly. The clinical model yielded AUCs of 0.712 (95% confidence interval [CI]: 0.6132 - 0.8106) and 0.706 (95% CI: 0.5480 - 0.8650) in the training and test sets, respectively.

**Table 2:** Univariate and multivariate logistic regression analysis of associated variables.

Characteristic	Univariate logistic regression analysis		Multivariate logistic regression analysis	
	OR(95%CI)	P-value	OR(95%CI)	P-value
Corticomedullary Enhancement Level	0.155(0.086-0.280)	p < 0.001	0.101(0.040-0.252)	p < 0.001
History of diabetes	0.263(0.115-0.602)	0.008	0.758(0.256-2.248)	0.675
Smoking history	0.346(0.183-0.654)	0.006	0.693(0.265-1.809)	0.529
Pseudocapsule	0.350(0.210-0.583)	0.001	0.650(0.291-1.454)	0.379
History of hypertension	0.366(0.223-0.601)	0.001	1.309(0.532-3.219)	0.623
Tumor location	0.381(0.235-0.618)	0.001	1.248(0.550-2.832)	0.657
Sex	0.393(0.260-0.595)	p < 0.001	0.604(0.223-1.637)	0.406
Cystic necrosis	0.489(0.322-0.744)	0.005	2.619(0.989-6.931)	0.104
Hematuria	0.625(0.245-1.597)	0.410		
Maximum Tumor Diameter	0.937(0.882-0.997)	0.085		
Lesion margin	0.950(0.561-1.610)	0.873		
Age	0.985(0.979-0.991)	p < 0.001	1.001(0.982-1.021)	0.921
Intratumoral vessels	1.000(0.595-1.682)	1.000		
Renal sinus involvement	1.158(0.691-1.939)	0.640		
Renal vein invasion	2.250(0.837-6.044)	0.177		

**Figure 4:**
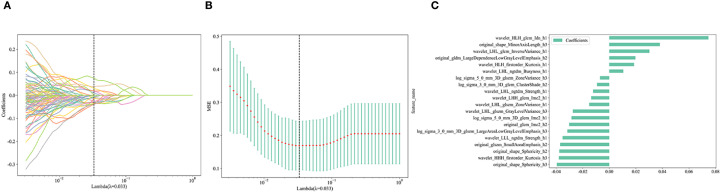
**(A)** The process by which the coefficients of all features shrink toward zero as the regularization parameter λ increases; **(B)** The optimal λ value was determined to be 0.033 via ten-fold cross-validation; **(C)** Selection of 20 optimal integrated habitat region radiomic features.

For conventional radiomics, 1,015 radiomic features were extracted from intratumoral and different peritumoral regions, including shape features (n = 14), first-order features (n = 198), gray-level co-occurrence matrix (n = 242), gray-level run-length matrix (n = 176), gray-level size-zone matrix (n = 176), neighborhood gray-tone difference matrix (n = 55), and gray-level dependence matrix (n = 154). Following feature selection (**Figure 4**), a five-fold cross-validation scheme was applied. For each region, the machine learning algorithm that produced the highest AUC on the test set was selected as the optimal classifier. Subsequently, the radiomics models were constructed using these selected algorithms.

**Figure 5:**
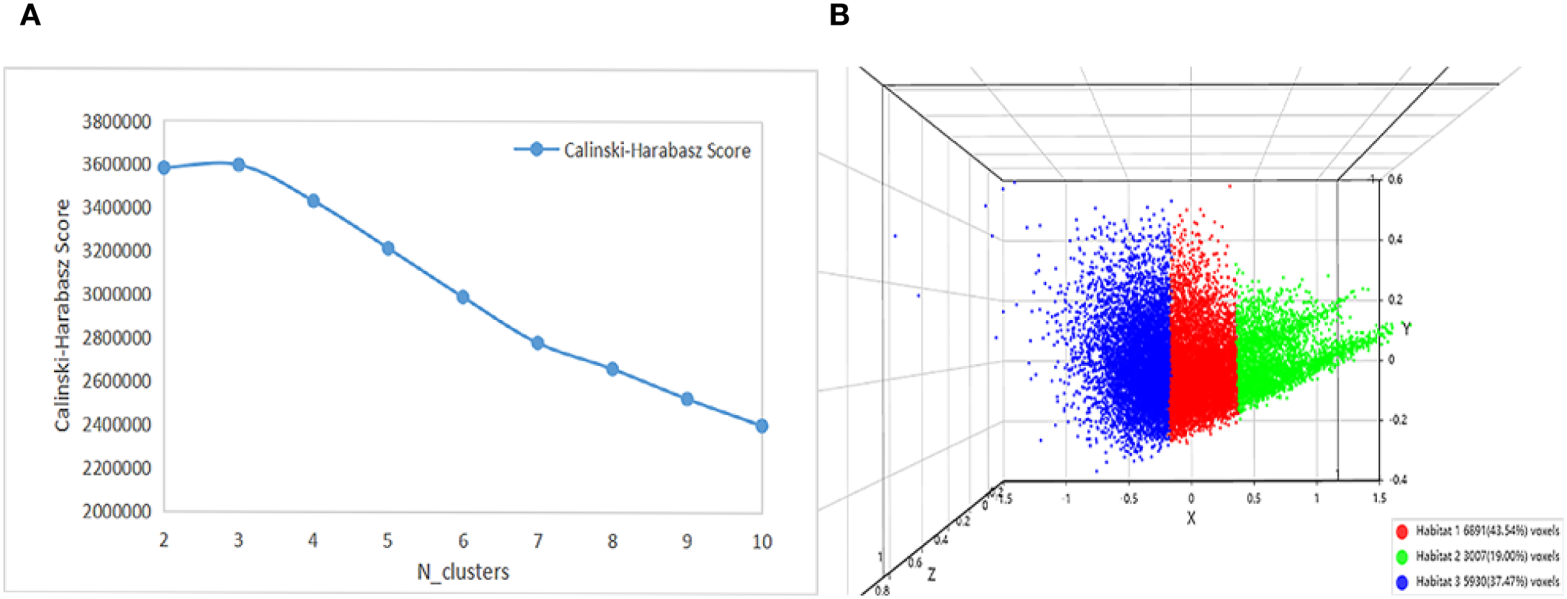
**(A)** Calinski-Harabasz scores across different cluster numbers. **(B)** Visualization of habitat clustering.

For habitat radiomics, the CH index determined 3 as the optimal cluster count. (**Figure 5A**), and the tumor was divided into three subregions. Clustering results were visualized using schematic diagrams generated from randomly selected 1% of voxels, with subregion voxel counts and proportions shown in **Figure 5B**. Each habitat subregion yielded 1,015 extracted features, and 3,045 features were obtained for the integrated habitat region. Thereafter, the same feature-selection workflow was applied to habitat features to build radiomic models for both the habitat subregions and the integrated habitat region.The optimal models for each region were determined as follows: Intra, Peri2mm, Peri5mm, HabitatH1, and HabitatH3 were best modeled by SVM; Habitat was best modeled by ExtraTrees; Peri1mm, Peri3mm, Peri4mm and HabitatH2 were best modeled by RF.

### 3.3 Comparison of model performance

**Figure 6:**
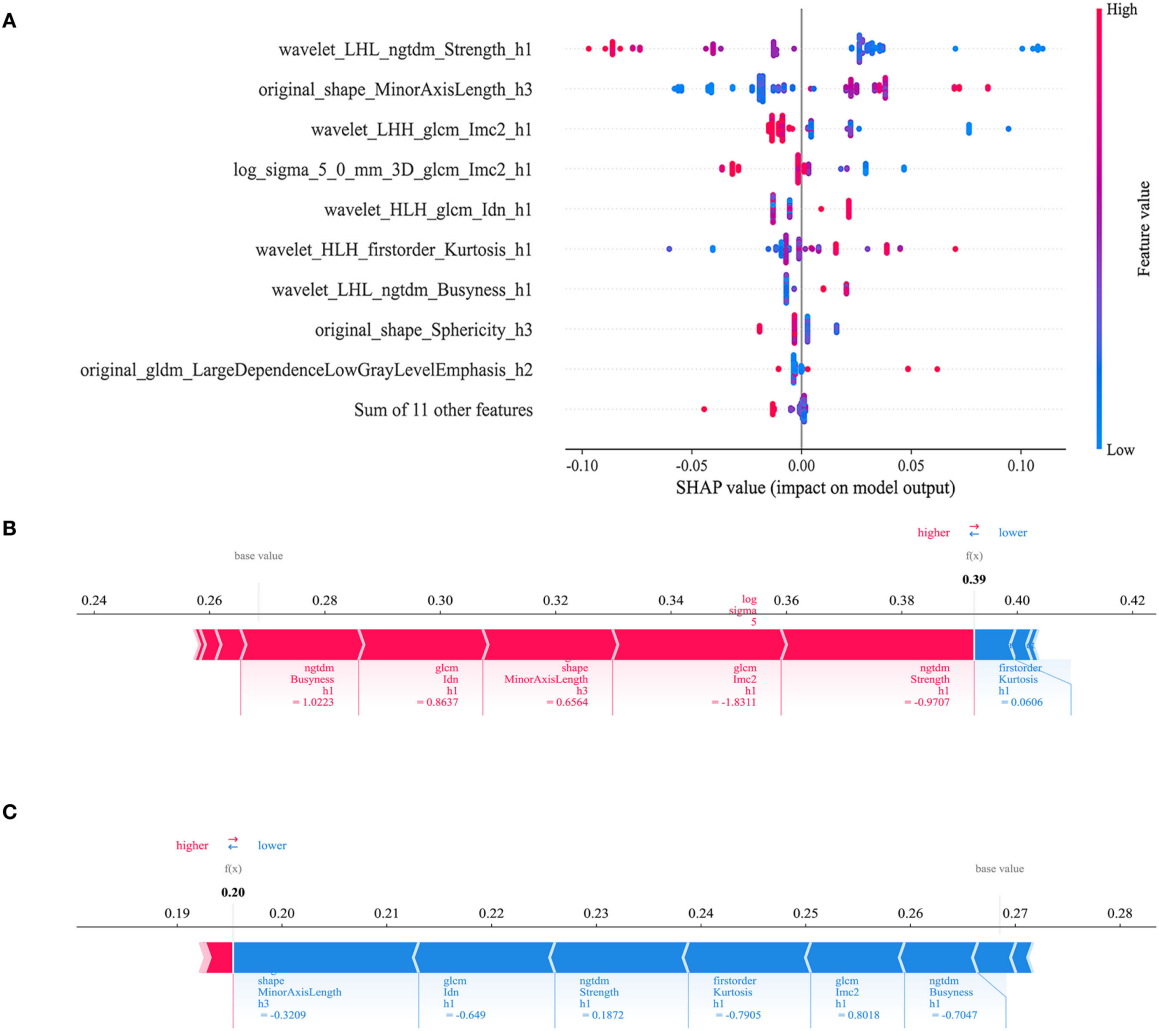
**(A)** SHAP bee swarm plot. It demonstrates the ranking of feature importance and their contribution to the prediction outcome. **(B, C)** SHAP force plots illustrate the discrimination mechanism of the Habitat model between high-grade and low-grade ccRCC cases.

The AUC for the Intra model was 0.848 (95% CI: 0.7622 - 0.9331) in the training set and 0.816 (95% CI:0.6285 - 1.0000) in the test set. Within the peritumoral regions, the Peri2mm model achieved AUCs of 0.884 (95% CI: 0.8187 - 0.9499) and 0.839 (95% CI: 0.7229 - 0.9550) in the training and test sets, respectively, outperforming other peritumoral extents and thus was selected for subsequent analyses. The Habitat model exhibited the highest AUC values in both the training set (AUC = 0.894, 95% CI: 0.8328 - 0.9547) and the test set (AUC = 0.877, 95% CI: 0.7642 - 0.9890), indicating strong discriminative capability compared with the habitat subregion models. Specifically, HabitatH1, H2, and H3 in the training and test sets had AUCs of 0.860 (95% CI: 0.7871 - 0.9322) and 0.851 (95% CI: 0.6942 - 1.0000), 0.869 (95% CI: 0.8033 - 0.9344) and 0.816 (95% CI: 0.6821 - 0.9491), and 0.858 (95% CI: 0.7760 - 0.9394) and 0.826 (95% CI: 0.6590 - 0.9930), respectively. The Habitat model also outperformed the Clinic model (AUC = 0.712 and 0.706), the Intra model (AUC = 0.848 and 0.816), and the Peri2mm model (AUC = 0.884 and 0.839). **Table 3** summarizes the comprehensive comparative results of all models.To analyze the decision-making mechanism of the habitat model, we employed SHAP for interpretability analysis. The SHAP swarm plot (**Figure 6A**) shows features ranked by contribution, with the x-axis representing SHAP values and the color of each point indicating the magnitude of feature values (red for high, blue for low). The most influential feature was wavelet_LHL_ngtdm_Strength_h1, where lower feature values corresponded to higher SHAP values, indicating a greater probability of predicting high-grade ccRCC. The SHAP force plot illustrates the individual prediction basis for two cases from the training set: for high-grade ccRCC, SHAP values were higher than the baseline (**Figure 6B**), while for low-grade ccRCC, SHAP values were lower than the baseline (**Figure 6C**). The key feature wavelet_LHL_ngtdm_Strength_h1 showed opposite contribution in the two cases (red arrows indicating increased probability of high-grade ccRCC, blue arrows indicating decreased probability), reflecting the model’s differentiated decision logic across various pathological contexts.

**Table 3:** Diagnostic performance of all models in the training set and test set.

Model	Cohort	AUC	95% CI	Accuracy	Sensitivity	Specificity	PPV	NPV
Clinic	train	0.712	0.6132 - 0.8106	0.722	0.690	0.734	0.488	0.866
Intra	train	0.848	0.7622 - 0.9331	0.852	0.793	0.873	0.697	0.920
Peri1mm	train	0.892	0.8288 - 0.9542	0.750	0.966	0.671	0.519	0.981
Peri2mm	train	0.884	0.8187 - 0.9499	0.806	0.931	0.759	0.587	0.968
Peri3mm	train	0.895	0.8388 - 0.9503	0.769	0.966	0.696	0.538	0.982
Peri4mm	train	0.845	0.7679 - 0.9227	0.778	0.724	0.797	0.568	0.887
Peri5mm	train	0.861	0.7927 - 0.9297	0.769	0.966	0.696	0.538	0.982
HabitatH1	train	0.860	0.7871 - 0.9322	0.824	0.759	0.848	0.647	0.905
HabitatH2	train	0.869	0.8033 - 0.9344	0.769	0.931	0.709	0.54	0.966
HabitatH3	train	0.858	0.7760 - 0.9394	0.806	0.793	0.81	0.605	0.914
Habitat	train	0.894	0.8328 - 0.9547	0.824	0.828	0.823	0.632	0.929
Combined	train	0.934	0.8830 - 0.9843	0.833	0.931	0.797	0.628	0.969
Clinic	test	0.706	0.5480 - 0.8650	0.696	0.727	0.686	0.421	0.889
Intra	test	0.816	0.6285 - 1.0000	0.870	0.727	0.914	0.727	0.914
Peri1mm	test	0.827	0.6699 - 0.9846	0.891	0.727	0.943	0.800	0.917
Peri2mm	test	0.839	0.7229 - 0.9550	0.696	1.000	0.600	0.440	1.000
Peri3mm	test	0.855	0.7114 - 0.9977	0.826	0.818	0.829	0.600	0.935
Peri4mm	test	0.836	0.7135 - 0.9592	0.739	0.909	0.686	0.476	0.96
Peri5mm	test	0.766	0.5968 - 0.9356	0.652	0.909	0.571	0.400	0.952
HabitatH1	test	0.851	0.6942 - 1.0000	0.848	0.727	0.886	0.667	0.912
HabitatH2	test	0.816	0.6821 - 0.9491	0.717	0.818	0.686	0.45	0.923
HabitatH3	test	0.826	0.6590 - 0.9930	0.848	0.818	0.857	0.643	0.937
Habitat	test	0.877	0.7642 - 0.9890	0.783	0.909	0.743	0.526	0.963
Combined	test	0.912	0.7875 - 1.0000	0.891	0.909	0.886	0.714	0.969

AUC, area under the curve; CI, credibility interval; NPV, negative predictive value; PPV, positive predictive value.

**Figure 7:**
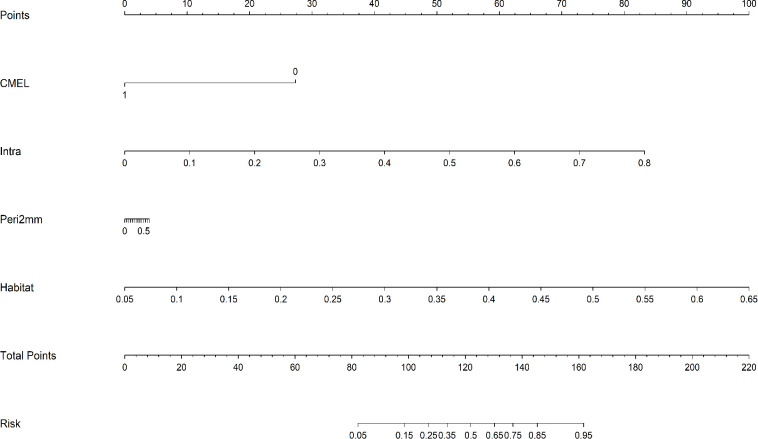
Nomogram for ccRCC patients.

**Figure 8:**
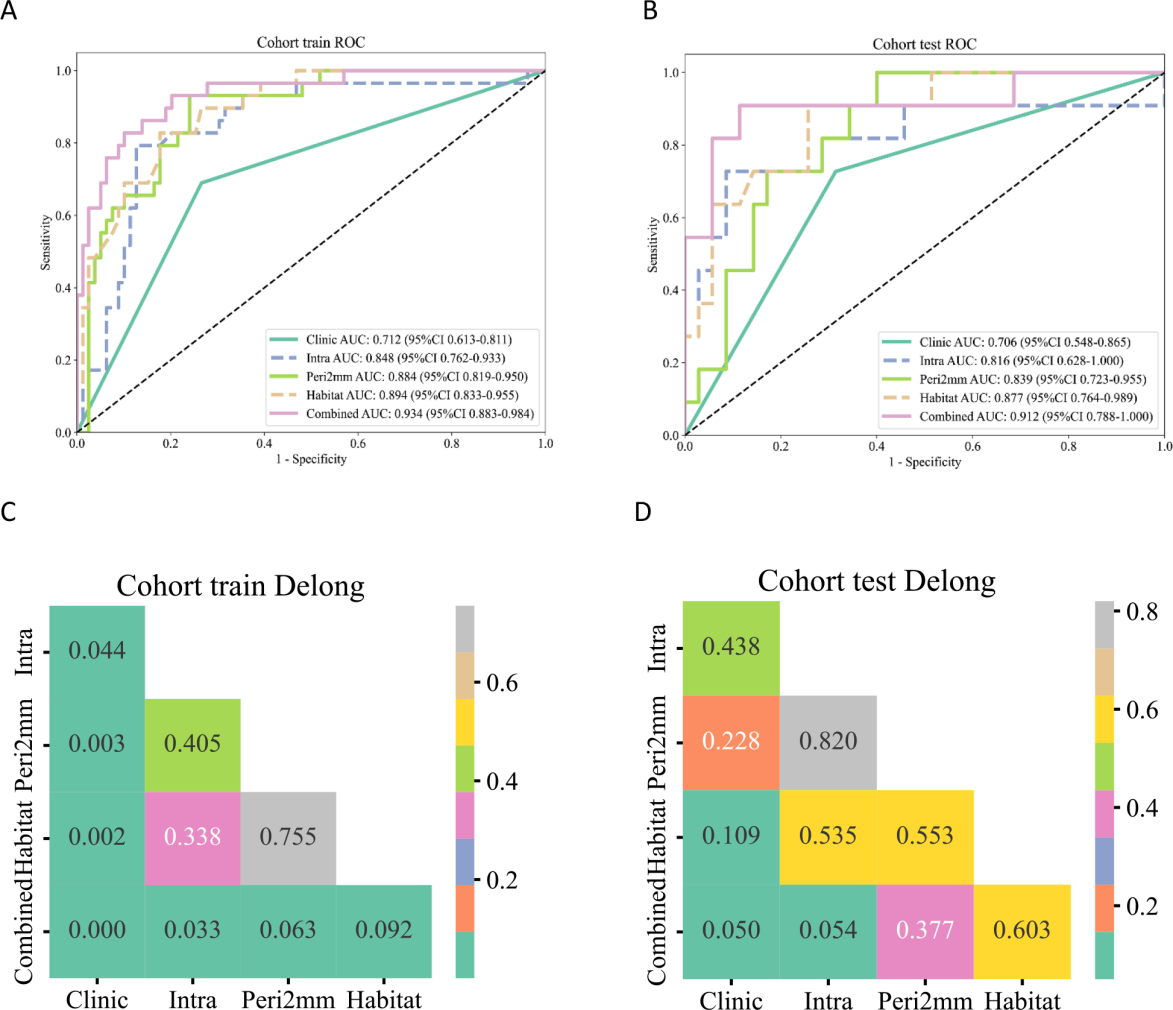
**(A, B)** ROC curves for different models. **(C, D)** Heatmap of DeLong test results.

**Figure 9:**
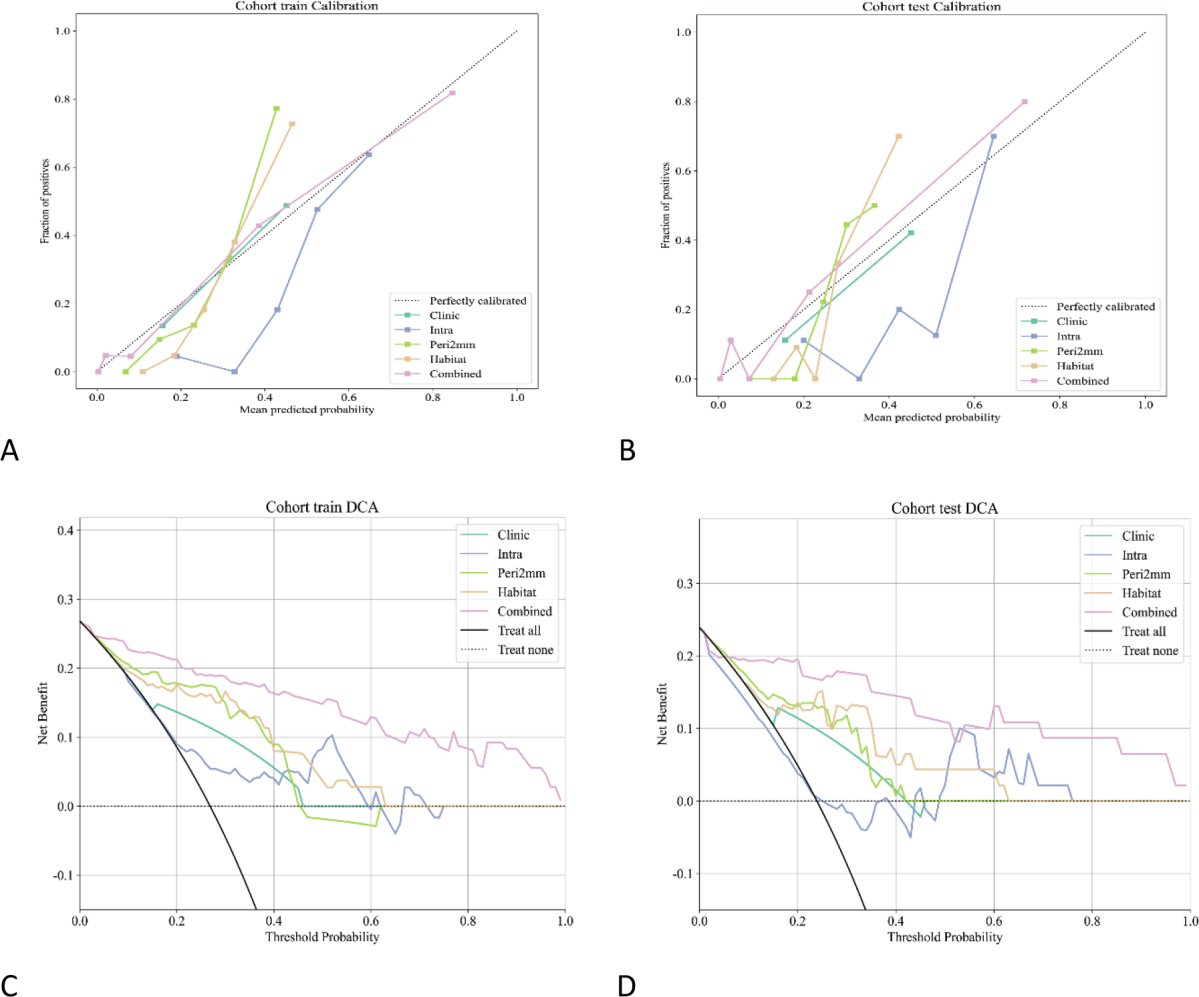
**(A, B)** Calibration curves for different models. **(C, D)** DCA curves.

Using corticomedullary enhancement level and features from the intratumoral, 2-mm peritumoral, and integrated habitat region, we constructed a combined nomogram model (**Figure 7**). This model achieved AUCs of 0.934 (95% CI: 0.8830 - 0.9843)in the training set and 0.912 (95% CI: 0.7875 - 1.0000) in the test set, significantly surpassing all individual models. A comparison of AUC values among the different models is shown in **Figures 8A, B**. DeLong’s test revealed that in the training set, the combined model significantly outperformed the clinical and Intra models (*P* < 0.05), while showing no significant difference compared with the Habitat and Peri2mm models (*P* > 0.05). In the test set, the combined model showed no statistically significant differences compared with the other models (*P* > 0.05) (**Figures 8C, D**). Calibration curves indicated satisfactory consistency between the nomogram-predicted probabilities and the observed outcomes (**Figures 9A, B**). AUC indicated that the combined model provided appreciable net clinical benefit across the full threshold range (0–1) in the training set, and across threshold ranges of 0.06–0.52 and 0.54–1.00 in the test set (**Figures 9C, D**). Although the 95% confidence interval in the test set was relatively wide (0.7875–1.0000), indicating some uncertainty in the performance estimate due to the limited cohort size, the lower bound remained at a relatively high level, suggesting that the model retains reasonably robust discriminative ability.

## 4 Discussion

This study presents the novel development of an MRI-based habitat radiomics model for predicting the WHO/ISUP nuclear grade in ccRCC, complemented by SHAP analysis to enhance interpretability. The results indicate that the Habitat model, by quantitatively capturing intratumoral heterogeneity, demonstrates improved discriminative performance compared with conventional approaches. Moreover, SHAP analysis helped identify key imaging features and dominant subregions potentially associated with nuclear grade. By integrating corticomedullary enhancement level with intratumoral, 2-mm peritumoral, and integrated habitat region features, we developed a combined nomogram model that demonstrated further improvement in predictive performance for nuclear grading and showed potential clinical utility. This study provides new insights into noninvasive nuclear grading for ccRCC and represents an important step toward precision medicine in the field of renal tumors.Previous studies on MRI-based radiomics have made significant progress in predicting nuclear grade in ccRCC (11, 12). Consistent with these studies, the intratumoral model developed in this study also demonstrated good performance for nuclear grading prediction, with AUCs of 0.848 and 0.816 in the training and test sets, respectively. Recent research has increasingly highlighted the importance of the peritumoral region, which represents the interface between the tumor and surrounding normal tissue and contains key biological information (15). The microenvironment of the peritumoral region is complex, consisting of endothelial cells, fibroblasts, immune cells, and extracellular matrix (16, 23). It is not only a critical site for tumor angiogenesis and lymphangiogenesis but also a major locus for immune cell infiltration and inflammatory responses, all of which contribute to tumor progression (15, 24, 25). High-grade ccRCC is highly invasive (26) and often leads to significant changes in adjacent tissue structures and morphology, which can be effectively captured by peritumoral imaging features (18), thus providing valuable information for noninvasive preoperative nuclear grading. Studies by Ma (17) and Li (13) have confirmed that incorporating peritumoral features improves the predictive performance of nuclear grading in ccRCC. They identified the optimal peritumoral regions as 2 mm and 5 mm, respectively. However, the optimal peritumoral range remains a topic of debate. In this study, we evaluated peritumoral ranges from 1 mm to 5 mm and found that the Peri2mm model performed best in both the training set (AUC = 0.884) and the test set (AUC = 0.839), which is consistent with Ma’s findings. This may suggests that the 2-mm peritumoral region offers a favorable balance between capturing microenvironmental features relevant to tumor nuclear grading and minimizing interference from distant normal tissue, thus optimizing both prediction accuracy and robustness. As the first systematic comparison of different peritumoral ranges, this study provides important evidence for determining the optimal peritumoral region for ccRCC nuclear grading prediction.

However, traditional intratumoral or peritumoral radiomics methods struggle to effectively capture the complex spatial heterogeneity within tumors. Emerging habitat imaging techniques, on the other hand, allow for the identification and quantification of subregions with similar imaging characteristics, enabling precise characterization of heterogeneous subregions. This provides a novel perspective for noninvasive analysis of tumor biological behavior and for further investigating the associations between tumor biology, histological features, treatment response, and clinical prognosis. For instance, Yuan et al. (14) developed intra- and peritumoral habitat models via multi-sequence MRI. Their study aimed to predict pathological complete response (pCR) in oral squamous cell carcinoma patients receiving immunotherapy. The study found that intratumoral and peritumoral habitat features could quantitatively represent the extent of CD45+ leukocyte infiltration in the tumor stroma and the expression level of PD-L1 in tumor cells. These immune microenvironment indicators were significantly correlated with pCR, thus validating the powerful capabilities of habitat imaging for noninvasively assessing tumor biological characteristics.

CT-based habitat radiomics has demonstrated value in predicting nuclear grade (27, 28), metastatic risk (29), and survival outcomes (30) in ccRCC, but MRI-based habitat radiomics studies remain largely unexplored. Given MRI’s superior soft-tissue resolution and multiparametric imaging capabilities, its application potential in ccRCC nuclear grading has yet to be thoroughly investigated. In this study, we employed voxel-level feature extraction, calculating 19-dimensional local radiomic features for each voxel. Three radiology-derived tumor habitat subregions were defined using CH-index-guided K-means clustering: Habitat 1 (moderate-to-high enhancement zone), Habitat 2 (non-enhancement zone), and Habitat 3 (transitional zone between Habitat 1 and 2). Habitat 1, which accounted for the largest proportion (43.54%), exhibited outstanding predictive capability compared with other subregions in both the training and test sets (AUCs = 0.860 and 0.851). Radiologically, this subregion corresponds to the area of most marked enhancement and likelyrepresents a core zone with active angiogenesis and high cellularity. Such characteristics may be associated with increased tumor aggressiveness, a higher risk of metastasis, and a poor prognosis. This observation was corroborated by SHAP analysis, which revealed that six of the top ten most important features originated from Habitat 1, further confirming the dominant role of this subregion in model decision-making. Owing to effective integration of features from all three habitat subregions, the Habitat model captured more comprehensive tumor information and achieved a test set AUC of 0.877, demonstrating better performance than the individual habitat subregion models and aligning with the findings of Lv (27) and Chen (28). Habitat features not only demonstrated stronger discriminative power than conventional radiomic descriptors but also produced models with superior overall performance.

This study identified the corticomedullary enhancement level as an independent predictor of ccRCC nuclear grade, further supporting previous reports (12, 31). It is postulated that high-grade ccRCCs grow rapidly and consequently outstrip their blood supply, leading to relative ischemia and pre-necrotic hypoperfusion that ultimately progresses to tissue necrosis. These alterations manifest as reduced overall enhancement, particularly evident in the corticomedullary phase. By integrating clinical variables with radiomic and habitat-derived features, the nomogram demonstrated outstanding discriminative performance in both the training and test sets (AUCs = 0.934 and 0.912, respectively), indicating its potential for stable, noninvasive preoperative grading. This relatively reliable predictive capability may hold significant implications for the clinical management of ccRCC. Accurate preoperative prediction of WHO/ISUP nuclear grade in ccRCC has important clinical implications. For tumors predicted as low grade (I–II), the model may serve as a valuable risk-stratification tool supporting more conservative management strategies, such as active surveillance or prioritization of nephron-sparing surgery, and may help identify patients for whom preoperative biopsy could be deferred or avoided. Conversely, lesions predicted as high grade (III–IV) may warrant earlier surgical intervention, more meticulous planning of the resection extent, and intensified postoperative surveillance. Thus, our findings provide an important, noninvasive, and potentially clinically useful adjunct to pre-treatment decision making for patients with ccRCC. Moreover, the optimal selection of surgical strategy remains a significant clinical challenge in individualized ccRCC management. This includes decisions regarding partial versus radical nephrectomy (32) and the choice of retroperitoneal or transperitoneal access (33). In the future, integrating noninvasive nuclear grade prediction with anatomical characteristics, such as tumor complexity and abdominal conditions, together with patients’ overall clinical status may facilitate the development of an integrated decision-support framework. Such an approach could provide a rational basis for personalized surgical planning and has the potential to improve perioperative management and long-term outcomes.

While this study has notable strengths, it also has certain limitations. (1) The retrospective, single-center design, combined with a limited sample size and an imbalanced distribution of high- and low-grade cases, may introduce selection bias and restrict the generalizability of the findings. Therefore, the present model’s clinical applicability should be interpreted with caution; future validation in larger, prospective, multicenter cohorts with multiparametric imaging is required to further substantiate model performance. (2) The model was developed using a single MRI sequence (contrast-enhanced T1-weighted imaging) and a single phase (the corticomedullary phase), with all data acquired on the same scanner model at a single institution with uniform imaging parameters. This homogeneity may limit the model’s performance when applied across centers and devices. Future work should integrate multi-sequence data (e.g., T2-weighted imaging, diffusion-weighted imaging, and apparent diffusion coefficient maps) and multiphase imaging (e.g., nephrographic, excretory phases) to capture a broader spectrum of tumor biology, and should validate the model on multicenter, multi-vendor cohorts to further evaluate and enhance its robustness and generalizability.(3) Manual regions of interest delineation is time-consuming and subject to interobserver variability, highlighting the need for automated tumor segmentation methods. Future work will focus on building a multi-omics tumor heterogeneity model and exploring the deep integration of habitat radiomics with artificial intelligence techniques. (4) The habitat partitioning in this study is a radiologic phenotyping result derived solely from MRI features and has not been validated against histopathology. Therefore, the precise spatial correspondence between each imaging-derived subregion and underlying biological structures (e.g., viable enhancing tissue versus necrotic tissue) remains undetermined and should be confirmed in future studies using image–pathology spatial co-registration techniques.

## 5 Conclusion

In conclusion, this study is the first to develop and validate a nomogram model based on MRI habitat radiomics for noninvasive preoperative prediction of the WHO/ISUP nuclear grade in ccRCC. By integrating clinical factors with intratumoral, peritumoral, and integrated habitat region radiomic features, the model demonstrates promising discriminative performance under internal validation. In addition, SHAP-based interpretability analysis was employed to clarify the model’s decision-making process, which may help increase confidence in the model’s predictions. In addition, SHAP-based interpretability analysis was employed to clarify the model’s decision-making process, which may help increase confidence in the model’s predictions This work furnishes preliminary feasibility evidence for noninvasive preoperative nuclear grading in ccRCC using MRI-based habitat radiomics and offers methodological guidance for the clinical translation of habitat radiomics. Nevertheless, the current model has only been preliminarily validated on single-center, single-sequence data; its clinical utility must be confirmed through external validation in larger, prospective, multicenter cohorts incorporating multiparametric imaging.

## Data Availability

The raw data supporting the conclusions of this article will be made available by the authors, without undue reservation.
