# Correction: MRI-based habitat radiomics for predicting WHO/ISUP nuclear grade in clear cell renal cell carcinoma

**DOI:** 10.3389/fonc.2026.1802556

**Published:** 2026-05-18

**Authors:** Naijing Shi, Cong Zhang, Xinyi Li, Mohan Hao, Chong Liu

**Affiliations:** 1 Department of Radiology, The First Central Hospital of Baoding, Baoding, Hebei, China; 2 Graduate School, Chengde Medical University, Chengde, Hebei, China

**Keywords:** clear cell renal cell carcinoma, habitat, peritumoral, radiomics, WHO/ISUP nuclear grade

There was a mistake in **Table 1** as published. “Test Set” was incorrectly written as “Training Set”. The numbers for the Test Set in **Table 1** were mislabeled: low-grade (n = 79) and high-grade (n = 29). The corrected **Table 1** appears below.

**Table 1:** Comparison of clinical features between the training set and test set.

Characteristic	Training set(n = 108)		P-value	Test set(n = 46)		P-value
	Low-grade	High-grade		Low-grade	High-grade	
	(n = 79)	(n = 29)		(n = 35)	(n = 11)	
Age	60.076 ± 10.793	63.862 ± 8.039	0.088	54.514 ± 12.832	59.818 ± 9.918	0.216
Maximum Tumor Diameter	3.756 ± 2.188	6.928 ± 2.342	<0.001	3.583 ± 1.450	8.182 ± 2.297	<0.001
Renal sinus involvement	0.241 ± 0.430	0.759 ± 0.435	<0.001	0.143 ± 0.355	0.818 ± 0.405	<0.001
Sex			0.788			0.464
Female	23 (29.114)	7 (24.138)		16 (45.714)	3 (27.273)	
Male	56 (70.886)	22 (75.862)		19 (54.286)	8 (72.727)	
Tumor location			1.000			0.066
Left	37 (46.835)	13 (44.828)		16 (45.714)	1 (9.091)	
Right	42 (53.165)	16 (55.172)		19 (54.286)	10 (90.909)	
Lesion margin			<0.001			0.052
Clear	59 (74.684)	10 (34.483)		26 (74.286)	4 (36.364)	
Unclear	20 (25.316)	19 (65.517)		9 (25.714)	7 (63.636)	
Smoking history			1.000			0.199
Absent	53 (67.089)	20 (68.966)		28 (80.000)	6 (54.545)	
Present	26 (32.911)	9 (31.034)		7 (20.000)	5 (45.455)	
History of hypertension			1.000			1.000
Absent	38 (48.101)	14 (48.276)		15 (42.857)	5 (45.455)	
Present	41 (51.899)	15 (51.724)		20 (57.143)	6 (54.545)	
History of diabetes			0.622			0.762
Absent	60 (75.949)	24 (82.759)		29 (82.857)	8 (72.727)	
Present	19 (24.051)	5 (17.241)		6 (17.143)	3 (27.273)	
Hematuria			0.501			0.427
Absent	71 (89.873)	24 (82.759)		31 (88.571)	8 (72.727)	
Present	8 (10.127)	5 (17.241)		4 (11.429)	3 (27.273)	
Pseudocapsule			1.000			1.000
Absent	39 (49.367)	15 (51.724)		17 (48.571)	6 (54.545)	
Present	40 (50.633)	14 (48.276)		18 (51.429)	5 (45.455)	
Intratumoral vessels			<0.001			<0.001
Absent	59 (74.684)	9 (31.034)		31 (88.571)	3 (27.273)	
Present	20 (25.316)	20 (68.966)		4 (11.429)	8 (72.727)	
Renal vein invasion			<0.001			0.002
Absent	75 (94.937)	20 (68.966)		34 (97.143)	6 (54.545)	
Present	4 (5.063)	9 (31.034)		1 (2.857)	5 (45.455)	
Cystic necrosis			0.092			0.281
Absent	32 (40.506)	6 (20.690)		11 (31.429)	1 (9.091)	
Present	47 (59.494)	23 (79.310)		24 (68.571)	10 (90.909)	
Corticomedullary Enhancement Level			<0.001			0.038
Lower than renal parenchyma	21 (26.582)	20 (68.966)		11 (31.429)	8 (72.727)	
Higher than or equal to renal parenchyma	58 (73.418)	9 (31.034)		24 (68.571)	3 (27.273)	

The original version of this article has been updated.

